# Modelling the molecular mechanisms of aging

**DOI:** 10.1042/BSR20160177

**Published:** 2017-02-23

**Authors:** Mark T. Mc Auley, Alvaro Martinez Guimera, David Hodgson, Neil Mcdonald, Kathleen M. Mooney, Amy E. Morgan, Carole J. Proctor

**Affiliations:** 1 Faculty of Science and Engineering, University of Chester, Chester, U.K.; 2 MRC/Arthritis Research UK Centre for Musculoskeletal Ageing (CIMA), Newcastle University, Newcastle upon Tyne, Ormskirk, U.K.; 3 Institute for Cell and Molecular Biosciences, Newcastle University, Newcastle upon Tyne, U.K.; 4 Musculoskeletal Research Group, Institute of Cellular Medicine, Newcastle University, Newcastle upon Tyne, U.K.; 5 Faculty of Health and Social Care, Edge Hill University, U.K.

**Keywords:** aging, computational models, computer simulation, modelling standards, molecular mechanisms

## Abstract

The aging process is driven at the cellular level by random molecular damage that slowly accumulates with age. Although cells possess mechanisms to repair or remove damage, they are not 100% efficient and their efficiency declines with age. There are many molecular mechanisms involved and exogenous factors such as stress also contribute to the aging process. The complexity of the aging process has stimulated the use of computational modelling in order to increase our understanding of the system, test hypotheses and make testable predictions. As many different mechanisms are involved, a wide range of models have been developed. This paper gives an overview of the types of models that have been developed, the range of tools used, modelling standards and discusses many specific examples of models that have been grouped according to the main mechanisms that they address. We conclude by discussing the opportunities and challenges for future modelling in this field.

## Introduction

Globally, the proportion of older people (aged 60 or above) is rising and it has been estimated that it will nearly double from 12% in 2015 to 22% by 2050 [1]. Furthermore, the ‘oldest-old’ (aged 80 or above) group is estimated to triple in the same time period. The increase in the aging population brings many challenges. Although many welcome the prospect of an increase in lifespan, this needs to be accompanied by an increase in healthy years rather than further years with disability and disease. Furthermore, there is a wide variation in the health among older individuals with some 80-year olds having the same physical and mental capacity of many 20-year olds, whereas other individuals experience a decline in physical and/or mental capacity at much earlier ages [1]. The reasons for this variability are complex and not understood. Therefore, there is an urgent need to increase our understanding of the underlying molecular mechanisms of the aging process, so that the continual increase in the proportion of older persons in the population will be beneficial rather than detrimental to future societies.

**Figure 1:**
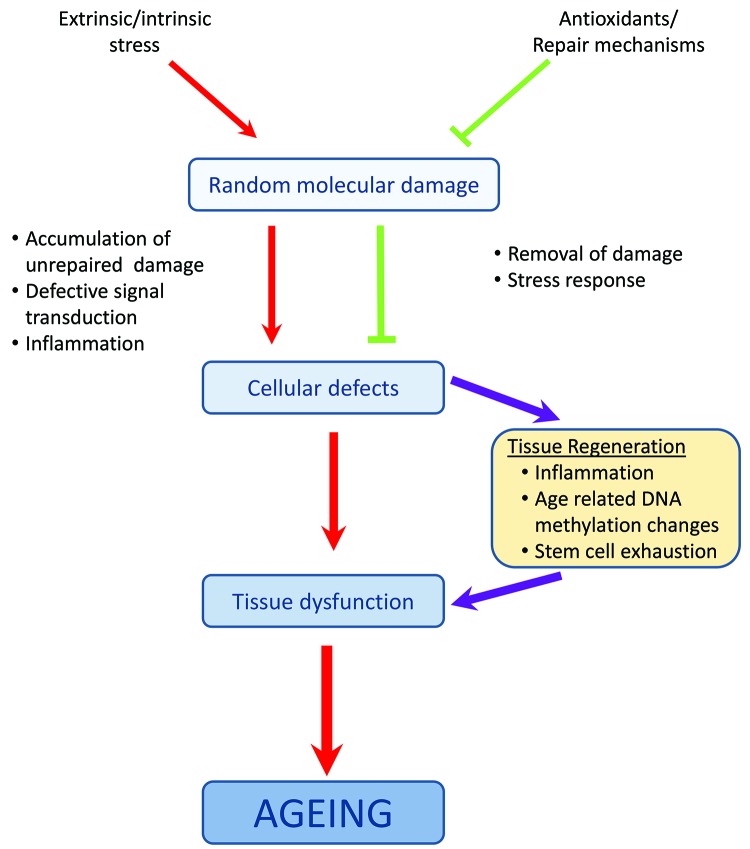
The underlying **mechanisms of aging** The rate of accumulation of stress-induced random molecular damage is dependent on the capacity of the antioxidant system and efficiency of repair systems. As these systems are not 100% efficient, cells always contain some unrepaired damage that leads to activation of a stress response and up-regulation of mechanisms to remove the damage or to prevent the cell division. However, these responses also become less efficient with age so that damaged components accumulate leading to cellular defects, which gives rise to tissue dysfunction and aging (redrawing of Kirkwood, T.B. [2]).

There are many theories of the biological causes of aging, which suggests that many different mechanisms contribute to the aging process [2,3]. Kirkwood proposed that the underlying cause is mainly due to the accumulation of random unrepaired molecular damage over time [2]. This eventually leads to cellular defects and tissue dysfunction resulting in increased frailty and age-related diseases [2], as illustrated in Figure 1. Our cells possess quality control systems so that molecular damage can be recognized, repaired or removed. However, due to the energy requirements of these systems, somatic maintenance is not 100% efficient. All molecular components are susceptible to damage including DNA, proteins, lipids and organelles. Sources of damage may be intrinsic, such as reactive oxygen species (ROS) and reactive nitrogen species (RNS) or extrinsic such as UV light, irradiation and exposure to toxins. In terms of aging, exposure to sources of damage over the human lifespan will vary among individuals and may in part explain the heterogeneity in how individuals age [4]. Other contributing factors include genetics, epigenetics, diet, physical activity and chance.

**Figure 2:**
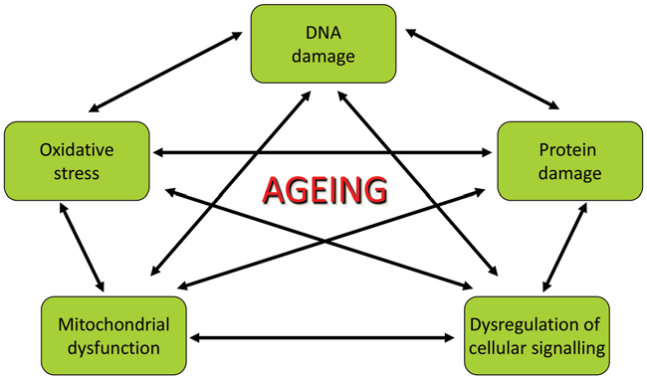
The **interaction of the molecular mechanisms of aging** Individual mechanisms cannot explain aging alone, as each mechanism has many interactions. Some example mechanisms and their interactions are shown but there are many others that are described in the text.

Many studies into the molecular mechanisms of aging have focused on a particular theory such as the accumulation of somatic mutations, telomere shortening, protein damage or mitochondrial dysfunction. However, in the late 1990s, it was realized that individual mechanisms cannot adequately explain the aging process [5] and that we needed to consider the interactions among these different mechanisms (Figure 2). For example damaged mitochondria produce more ROS that in turn leads to an increase in damage to all molecular components. This led to a network theory of aging and the challenge of studying complex interactions motivated one of the first integrated mathematical models of aging [5]. Since then, the advent of many new technologies and the ability to produce large volumes of experimental data necessitated the development of new tools to aid analysis and interpretation, leading to the emergence of systems biology approaches [6–8].

Despite these advances, many experimental biologists remain unaware or sceptical of the use of computational models as a research tool. Therefore, in this review, we give a brief explanation of the advantages of this approach to study complex biological systems. Firstly, models can be used to test hypotheses and as every component and the interactions among components need to be clearly defined, it ensures that hypotheses are specific. Secondly, models can highlight gaps in knowledge, particularly during the model-building process. Thirdly, models can make predictions that can then be tested experimentally and so help to advance our understanding of complex systems. Lastly, models can be used to test multiple potential interventions, which would be very costly and time consuming to do experimentally.

The purpose of this review is to give an overview of mathematical and computational models that have been developed to examine the molecular mechanisms of aging. We will start by describing some of the different computational approaches including types of models, modelling standards, software tools and a summary of models that are publicly available from the BioModels database [9] (see section on ‘Modelling standards’). We will then describe in more detail, some of the models that have been developed over the last 15 years of particular molecular mechanism including DNA damage, loss of protein homoeostasis, mitochondrial dysfunction, dysregulation of cellular signalling pathways and epigenetic changes with age. Finally, we highlight some of the challenges and opportunities for future research, in which we discuss integration of mechanisms and multi-scale modelling.

## Computational approaches

### Types of models

The formalization of a computational model begins with the evaluation of a series of fundamental questions. The first one addresses if the model is a static or a dynamic representation. Static models, commonly constructed through network inference algorithms, are primarily concerned with establishing statistical relationships among biological entities [10–13]. Static models are commonly used in the identification of structural and functional patterns in large bodies of ‘omics’ data [14–16] and for comparative studies [10].

Dynamic models aim to capture how the variables of interest evolve over time [17]. This contrasts with the ‘snapshot representation’ of static models [15]. However, dynamic models commonly require a comparatively larger number of parameters in order to capture behaviour along the time dimension [13]. Because biological research inevitably concerns processes that occur over time, dynamic models are the most intuitive option to represent a biological system.

The second fundamental question addresses the very nature of the process being modelled. Is it a discrete process or a continuous process? Alternatively, how well can a given process be approximated as being discrete or continuous whatever its underlying nature? A discrete process involves a series of identifiable states of the observable of interest, for example the number of molecules in a cell or the number of fish in a pond. In a continuous process, the solution space cannot be divided into discrete observables, for example the joules of energy produced by the electron transport chain.

The third fundamental question involves the role of randomness or stochasticity, in the biological process. In a system that is not significantly affected by noise, deterministic models, which invariably show the same behaviour per given parameter set and initial conditions, can be used [18]. Deterministic frameworks are commonly employed in the modelling of mechanical systems. In noisy systems, however, there is an intrinsic uncertainty in the relationship between parameters [18,19]. For example whether a reaction occurs at a given moment in time depends on the probability of the encounter of the substrates in space. Stochastic models involve sampling from probability distributions to account for this uncertainty. The Gillespie algorithm [20], for instance is a powerful method to model biochemical networks stochastically [19]. This is because it captures the uncertainty associated with which reaction will occur next in a cell and when exactly it will take place.

**Figure 3:**
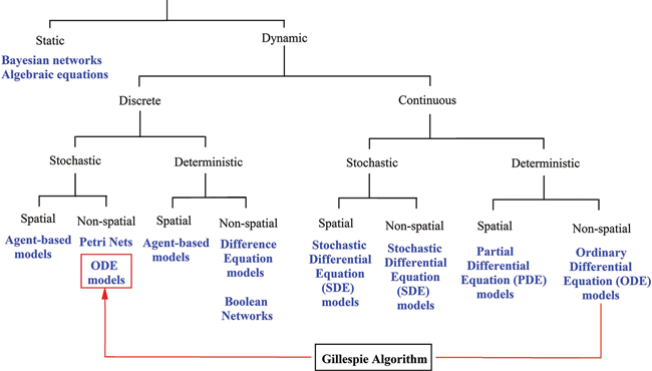
A pragmatic classification of modelling frameworks The first decision concerns whether the model must capture the behaviour of the system (Dynamic) or only its structure (Static). Because aging, health and disease are processes, dynamic modelling of biological systems is a common approach within computational modelling. The second decision addresses whether the time-evolving behaviour of the system can be broken down into discrete states (Discrete) or not (Continuous). Within both of these partitions, a model can have fixed trajectories for a given parameter set and initial conditions (Deterministic) or contain a degree of uncertainty that makes it probabilistic in nature (Stochastic). Within both of these approaches, one can account for the spatial dimension if deemed appropriate. Examples of commonly employed computational frameworks for each classification are shown in blue. Note that the development of many frameworks has resulted in the transcending of the traditional classification boundaries. Examples include stochastic Boolean networks or dynamic Bayesian networks. An important consideration is how well the biological system can be approximated by a given modelling framework, regardless of its underlying fundamental nature. This is exemplified by the Gillespie algorithm, which can simulate continuous-deterministic ordinary differential equation (ODE) models as discrete-stochastic models given a previous adjustment of rate constants and a unit conversion to particle numbers. Another example would be the conversion of continuous models from deterministic to stochastic by the addition of a noise factor to the differential equations. For a more detailed description of these and other modelling frameworks, see [23,24]. Within the technical realm, modelling frameworks can be broadly classified into mathematical models, algorithmic models and hybrid models [25].

The last fundamental consideration is whether the behaviour of the process modelled is dependent on the spatial dimension as well as the time dimension. An example of this would be the effect of intracellular gradients on cellular signalling [21] or localized niches in ecological studies [22]. Figure 3 displays the conceptual classification of models and the common frameworks used in each classification.

### Modelling tools

There are a plethora of software tools that can be used to construct an *in silico* model of a biological system. These software tools fall into two broad categories: (i) commercial tools, which include packages such as Mathematica, Matlab and Maplesim and (ii) non-commercial open source software, which include software such as R, Python and C++. For experienced modellers, the choice is dependent on personal preference. However, fortunately for a biologist unfamiliar with modelling, the last decade has witnessed the development of several tools that are exceptionally user-friendly. Examples include Copasi [26] and CellDesigner [27], both of which are underpinned by intuitive user interfaces that allow models to be assembled by the addition of reactions as word equations. The user then selects the type of kinetic rate law that is appropriate for each reaction from a drop-down menu. To run a deterministic simulation, the software tool converts the reaction list to a set of coupled ODEs, which are numerically solved to provide model output. A stochastic model is assembled in a similar fashion, with rate laws replaced by propensity functions. The software tool then uses the Gillespie algorithm or one of its derivatives to perform stochastic simulations of the model.

### Modelling standards

Regardless of the software tool that is utilized, systems biology models need to be accessible and straightforward to update and extend. This is particularly important for aging, as our knowledge of this phenomenon continues to evolve. To facilitate this aspect of modelling, the computational systems biology community have developed a number of exchange frameworks. For instance PySB is a framework for building mathematical models of biological systems using Python [28]. However, the leading model exchange format is the systems biology markup language (SBML) [29]. A large number of modelling tools support this framework and a full list is provided on the SBML website (http://sbml.org/SBML_Software_Guide/SBML_Software_Summary). It is a good modelling practice to submit an SBML-encoded model for archiving in BioModels [9]. BioModels is an online database (https://www.ebi.ac.uk/biomodels-main/) that stores the details of a model and assigns each model a unique identification number. Within this repository, a model can be categorized as either curated or non-curated. Curated models have been verified so results match its corresponding publication, whereas non-curated models await this process.

### Aging-themed models archived in BioModels

There is a wide variety of aging-focused models stored in BioModels. We surveyed BioModels using the search terms ‘ageing’ and ‘aging’ and found 22 in the curated and 6 in the non-curated section (Tables 1 and 2). These models cover many aspects of molecular aging. It is not possible to discuss each model, however within the context of this review, a number of models are worth highlighting. The model by Dalle Pezze et al. [30] is particularly noteworthy due to the close coupling of experimental work with model simulations. This model was able to consolidate experimental findings, which demonstrated that inhibition of ROS can inhibit the loss of mitochondrial membrane potential (BIOMD0000000582). The work of Geva-Zatorsky et al. [31] encapsulates another important element of molecular aging, the p53 system, while also juxtapositioning experimental work with computational modelling. The authors quantified the dynamics of fluorescently tagged p53 and Mdm2 over several days and found that isogenic cells in a homogeneous environment behaved in a highly stochastic manner following DNA-damaging γ-irradiation and cells showed oscillations over several days. To explore this phenomenon in greater depth, the authors used different mathematical models of the system to identify the source of the oscillations, which was revealed to be low-frequency noise in protein production rates, rather than noise in other parameters, such as degradation rates (BIOMD0000000154 to BIOMD0000000158). A more recent cellular model by Erguler et al. [32] uncovered three unique states of behaviour; low, high and intermediate activity, which were correlated with stress adaptation, resistance and the initiation of apoptosis (BIOMD0000000446). Collectively, these examples give an overview of the diversity of cellular processes associated with aging, which have been modelled and are archived in BioModels.

**Table 1:** Curated models with an aging theme archived within BioModels

Model	BioModel ID
A quantificative model of the generation of N(ε)-(carboxymethyl)lysine in the Maillard reaction between collagen and glucose	BIOMD0000000053
Modelling the actions of chaperones and their role in aging	BIOMD0000000091
Alternative pathways as mechanism for the negative effects associated with overexpression of superoxide dismutase	BIOMD0000000108
A mathematical model of glutathione metabolism	BIOMD0000000268
Experimental and computational analysis of polyglutamine-mediated cytotoxicity	BIOMD0000000285
Feedback between p21 and reactive oxygen production is necessary for cell senescence	BIOMD0000000287
A mathematical model of the unfolded protein stress response reveals the decision mechanism for recovery, adaptation and apoptosis	BIOMD0000000446
*In vivo* and *in silico* analysis of PCNA ubiquitylation in the activation of the post replication repair pathway in *S. cerevisiae*	BIOMD0000000475
Feedback motif for the pathogenesis of Parkinson’s disease (PD)	BIOMD0000000558
A model of the coupling among brain electrical activity, metabolism and haemodynamics: application to the interpretation of functional neuroimaging	BIOMD0000000570
Simulated interventions to ameliorate age-related bone loss indicate the importance of timing	BIOMD0000000612
Modelling the checkpoint response to telomere uncapping in budding yeast	BIOMD0000000087
Modelling the actions of chaperones and their role in aging	BIOMD0000000091
An *in silico* model of the ubiquitin-proteasome system that incorporates normal homoeostasis and age-related decline	BIOMD0000000105
Explaining oscillations and variability in the p53–Mdm2 system	BIOMD0000000188
Explaining oscillations and variability in the p53–Mdm2 system	BIOMD0000000189
A whole-body mathematical model of cholesterol metabolism and its age-associated dysregulation	BIOMD0000000434
Aggregation, impaired degradation and immunization targeting of amyloid-β dimers in Alzheimer’s disease (AD): a stochastic modelling approach	BIOMD0000000462
Investigating interventions in AD with computer simulation models	BIOMD0000000488
Mathematical modelling of cytokine-mediated inflammation in rheumatoid arthritis	BIOMD000000054
Oxidative changes and signalling pathways are pivotal in initiating age-related changes in articular cartilage	BIOMD0000000560
Dynamic modelling of pathways to cellular senescence reveals strategies for targeted interventions	BIOMD0000000582

**Table 2:** Non-curated models with an aging theme

Model	BioModels ID
Mathematical modelling for the pathogenesis of AD	MODEL1409240001
Modelling of calcium dynamics in brain-energy metabolism and AD	MODEL1409240003
To senesce or not to senesce: how primary human fibroblasts decide their cell fate after DNA damage	MODEL1505080000
Modelling the response of FOXO transcription factors to multiple post-translational modifications made by aging-related signalling pathways (Pathways A–C)	MODEL1112260000: Pathway A MODEL1112260001: Pathway B MODEL1112260002: Pathway C

## Models of molecular mechanisms of aging

In this section, we describe some of the previous models that have been developed to examine the molecular mechanisms of aging (See each individual section below for the citations of the models). Many of these models focus on a particular mechanism and thus are categorized according to mechanism. However, due to the interaction of mechanisms, some models that are placed in a particular category will also contain mechanisms from another e.g. a model of protein aggregation also incorporates the DNA damage response (DDR).

### DNA damage and repair

The accumulation of unrepaired DNA damage has long been proposed as a major causal factor in aging (reviewed in [33]). DNA is susceptible to damage due to replication errors, intrinsic stress due to ROS and extrinsic stress such as UV light and irradiation. Most damage to DNA is detected and repaired via the DDR involving ATM and p53 signalling. However, more complex lesions may remain unrepaired and accumulation of such lesions may lead to apoptosis, cellular senescence or cancer. Many models have examined the role of DNA damage on cellular senescence based on cells in culture such as human fibroblasts [34–37]. These models did not include details of the molecular mechanisms involved in the DDR, in which cellular signalling pathways involving ATM/ATR, p53 and p21 are activated, resulting in cell cycle arrest to allow for possible repair. DNA damage induced by irradiation causes levels of p53 and its inhibitor Mdm2 to oscillate [38], and it was shown by mathematical models, that this behaviour was due to negative feedback loops in the system [31,39–41].

It has been shown experimentally that ROS acts in a positive feedback loop to activate and maintain cells in replicative senescence and this was confirmed by mathematical modelling [42]. This model of DDR was an extension of a previous model of p53 dynamics [41], demonstrating the advantage of using SBML for model construction. In addition, the extended model showed that stochastic effects are important as it predicted that a small proportion of cells could escape cell cycle arrest, which was then later confirmed experimentally [42].

Models of DNA repair pathways have mostly been motivated by the need to improve cancer therapies [43,44]. Using experimental data of human fibroblasts that had been subjected to different levels of γ-irradiation, a stochastic model of the non-homologous end joining (NHEJ) pathway showed the importance of redox regulation of the proteins Ku70 and Ku80, which form heterodimers that bind to the ends of DNA double-strand breaks [45]. The authors of this model, then went on to integrate their model with the DDR model previously developed by Passos et al. [42], producing the first model to incorporate both the DDR and DNA-repair pathways [46]. Although both the previous models were encoded in SBML and therefore very amenable to integration, the use of stochastic simulation for the integrated model proved infeasible. Therefore, the model was converted into a rule-based system using BioNetGen. The model was used to examine the effect of irradiation on DNA-damage repair and induction of senescence.

### Telomere shortening

Telomeres are repetitive sequences of DNA that protect the ends of linear chromosomes. In human somatic cells, telomeres shorten with each cell division due to the end-replication problem and it has been suggested that telomere shortening explains why human diploid fibroblasts can only divide a limited number of times (known as the Hayflick limit) before a cell undergoes replicative senescence [47]. The first mathematical models focused on this process [48–51], but it was later shown experimentally that oxidative stress is an important factor, contributing to an increase in the telomere shortening rate by up to an order of magnitude [52]. This additional factor was included in the models of Rubelj and Vondracek [53] and Proctor and Kirkwood [54]. The latter model also included free-radical species as a model parameter and helped to explain why cells cultured under conditions of increased oxidative stress have reduced replicative capacity. The models of telomere shortening so far described assumed that either a critically short telomere or a subset of short telomeres triggered replicative senescence. However, it has been shown that cells stop dividing over a wide range of telomere lengths. Since telomeres are protected by various telomere-binding proteins and the formation of T loops, it has been hypothesized that the disruption (uncapping) of these loops may be the actual trigger. The model by Proctor and Kirkwood [54] was adapted so that the trigger for senescence was an uncapped telomere, with the assumption that the probability of uncapping increases as telomere length shortens and this model provided a better fit to the experimental data [55]. Rodriguez-Brenes and Peskin [56] modelled the biophysics of the T loop whereby the T loop represented the capped state and also included telomerase, an enzyme that elongates telomeres in non-somatic cells such as germ-line cells. Telomere shortening alone is unlikely to account for the observed heterogeneity in the doubling potential of cells from within the same clone [57]. This was elegantly shown by an integrated model, which incorporates not only telomere shortening but also nuclear somatic mutations and mitochondrial damage [58]. On a rather different track, Aviv et al. [59], proposed a model linking telomere dynamics with successful compared with unsuccessful aging based on evidence that individuals with short telomeres have a higher risk of atherosclerosis [60].

### Loss of protein homoeostasis

Protein homoeostasis is crucial for cellular function and is maintained by quality control systems involved in protein synthesis, folding and refolding (reviewed by [61]). Evidence for the role of damaged proteins in aging is the observed increase in oxidized proteins with age in brain [62]. Proteins are susceptible to oxidative damage that results in either conformational or covalent changes. Most forms of covalent damage are irreversible and so proteins with such damage need to be degraded in order to prevent their accumulation and cross-linking. Conformational damage may be repaired by molecular chaperones that bind to the exposed hydrophobic surfaces and assist in refolding. However, it has been proposed that the chaperone system becomes overwhelmed with age leading to a further increase in damaged or misfolded proteins [63].

### Molecular chaperones

Molecular chaperones are up-regulated during stress by a feedback mechanism involving the transcription factor heat shock factor-1 (HSF-1) and the molecular chaperone Hsp90 [64]. Normally, HSF-1 is kept in an inactive monomeric state by binding to Hsp90. However, under stress conditions, there is an increase in misfolded proteins that also bind to Hsp90 releasing HSF-1, which can then trimerize, translocate to the nucleus and become transcriptionally active. This results in an increase in molecular chaperones that can then assist in refolding the denatured proteins. Several models of the heat-shock response in response to elevated temperature have been developed that have used a deterministic approach e.g. [65,66]. However, to date, only two models have included the effects of aging on the system [67,68]. Proctor et al. [67] included a mechanism for an increase in misfolded protein with age as a result of increased oxidative stress and also examine the effect of damage to the molecular chaperones themselves. Since damage is a random process, they used stochastic simulation. The model showed that the chaperone system was able to maintain homoeostasis under conditions of mild or transient stress. However, chronic stress eventually led to a point when the balance between molecular chaperones and misfolded proteins could not be maintained, so the misfolded proteins bound together to form aggregates. This model was extended to include the chaperone Hsp70 and its role in apoptosis, which allowed for the possibility that cells with high level of misfolded proteins may undergo programmed cell death [68].

### Protein degradation pathways

Proteins are constantly turned over in the cell although there is large variability in the half-lives of different proteins. There are two main pathways for protein degradations, the autophagic system and the proteasomal system. Both systems may eliminate damaged proteins. Oxidized proteins may be eliminated by 20S proteasome [69] or the ubiquitin/proteasome system (UPS) [70], but their efficiency declines with age leading to a build up of damaged protein especially in post-mitotic cells [61]. It has been hypothesized that damaged proteins overwhelm the capacity of proteasomes and that coupled with age-related damage to the proteasome results in a self-amplifying cycle of impairment [71]. Previous mathematical models of protein degradation by the proteasome have examined the kinetics of peptide hydrolysis (reviewed in [72]) but very few models exist that examine the role of protein degradation in the context of aging. A model of the ubiquitin–proteasome system that incorporated normal homoeostasis and age-related decline was developed by Proctor et al. [73]. In addition to the effects of an increase in damaged and aggregated protein, depletion of ubiquitin pools was shown to also contribute to the decline in protein homoeostasis with age.

The autophagic system includes macroautophagy and chaperone-mediated autophagy (CMA), which both functionally decline with age [74]. CMA is up-regulated by oxidative stress in order to degrade damaged proteins [75]. It is inhibited by mutant proteins such as modified α-synuclein [76]. There is cross-talk between autophagy and apoptotic pathways, and a mathematical model was used to examine how levels of stress determine the switch between these two outcomes [77]. Despite the lack of models on the effects of aging on protein degradation, several models of protein aggregation in age-related neurodegenerative disorders such as AD and PD, also include protein degradation pathways and will be discussed below.

### Protein aggregation

Many models of protein aggregation in age-related neurodegenerative disorders have been developed [78–85]. Some of the models focus just on the dynamics of the aggregation process (reviewed by [86]), whereas others incorporate other processes such as protein degradation [81,87,88], chaperones [89], neuroinflammation [90,91], the DDR [92], oxidative stress [80] and apoptotic pathways [82]. The aggregation of α-synuclein is a key factor in PD. It is degraded by macroautophagy, CMA or the proteasome and is susceptible to modifications, which make it prone to aggregate. This complex system has motivated several models that have incorporated both autophagic and proteasomal pathways to examine the mechanisms involved in α-synuclein aggregration [80,81,88,93]. Raichur et al. [80] also included oxidative stress in their model and showed that α-synuclein aggregation increased with stress supporting the hypothesis that an increase in oxidative stress may precipitate sporadic PD (reviewed by [94]).

Many models of the aggregation process in AD have also been developed. Two different aggregates are implicated, namely amyloid plaques and τ tangles, however, the majority of models focus only on the aggregation of amyloid-β e.g. [79,84,90,91]. Surprisingly, very few models consider the aggregation of τ despite many unanswered questions regarding the relative contribution of plaques and tangles to disease progression and the mechanisms that link the two aggregation processes. The only models that we are currently aware of are all from one group [85,92] and further modelling suggested that there is a cycle of events, which can explain why plaques, tangles or both are seen in the aging brain [95].

The majority of models examine the aggregation process based on short timescales based on *in vitro* cellular models. Computer simulation time also limits timescales for stochastic models. However, by making simplifying assumptions, it was possible to simulate aggregation of amyloid-β over a 100-year period [84] and to use this model to test intervention strategies.

Although mitochondrial function has also been shown to play an important role in age-related neurodegeneration, there are currently no models of protein aggregation that explicitly include mitochondria. Since mitochondrial dysfunction leads to an increase in oxidative stress, impaired energy and inhibits autophagy, linking models of mitochondrial damage and ROS (see next section) and protein aggregation would provide new insights into the causes of age-related decline in protein homoeostasis.

### Mitochondrial damage and ROS

Ever since Harman expanded his free radical theory of aging [96] to include mitochondria [97], the main site of ROS production in cells, abundant evidence has amounted on the association of both increased ROS levels and dysfunctional mitochondria with age and age-related diseases [98–102]. The complexity of mitochondria as an organelle and the short-lived nature of ROS molecules have resulted in experimental difficulties in dissecting a potential causal role in the driving of the aging process [103–105]. However, computational modelling efforts have laid some important conceptual and mechanistic groundwork over which experimental methodologies may build upon despite their current limited resolution.

Kirkwood and Kowald [106] have made use of a variety of models to conceptually establish some phenomena that could be causative of the observed phenotypes of mitochondrial dysfunction. They demonstrated how increased antioxidant expression in mitochondria is unlikely to reduce damage to mtDNA [106]. They furthermore discriminated among different potential driving mechanisms of mitochondrial heteroplasmy, where a faster transcription was established to be a source of selective advantage for mtDNA mutants rather than random drift alone or a smaller genome size [107,108]. Another example of laying conceptual groundwork through computational modelling comes from the work of Lawless et al. [109]. The authors established that the continuous increase in ROS levels seen in cultured cells undergoing senescence can be explained by a stochastic entry of individual cells into a senescent state with a time-invariant ROS level.

Computational models have also been used to formalize mechanistically the homoeostatic mechanisms regulating both ROS levels and mitochondrial function [110–113]. Gauthier et al. [114] developed a comprehensive computational model that captures mitochondrial ROS production in different mitochondrial energetic states as determined by pacing frequency. Their model established a link between dysfunctional calcium handling and increased ROS production by the mitochondria due to the elevated cytosolic sodium levels seen in aged cardiomyocytes [114]. While Passos et al. [42] used computational modelling to prove that the driving of the cellular senescence state involves ROS production by dysfunctional mitochondria, Dalle Pezze et al. [30] constructed the first comprehensive model of cellular senescence. They reported an increased stochasticity and reduced network sensitivity to both endogenous signals and exogenous treatments. These higher order observations on network-wide changes during senescence are coherent with the accepted notion on aging being a holistic, multi-mechanism process [6,115].

### Mitochondrial dynamics

Aging is associated with the accumulation of damaged mitochondria, which may be due to a decline in mitochondrial turnover. There have been a number of computational models that have investigated this phenomenon. For instance the model developed by Kowald and Kirkwood [116] was used to demonstrate that damaged mitochondria have impaired energy metabolism. The model also showed that as a compensatory mechanism, mitochondria have a reduced rate of degradation, which results in clonal expansion of damaged mitochondria. This theoretical work was able to consolidate experimental evidence that aged muscle fibres often contain a reduced number of mtDNA mutant types. Simulations also suggested that cellular division can rejuvenate and stabilize the mitochondrial population, in accordance with experimental data that suggest that mitochondrial damage accumulates faster in post-mitotic tissues than mitotically active tissues. Importantly, the authors suggest that *in vitro* studies of aging underestimate the contribution of mitochondrial-related cell degradation to cell aging [116]. This finding is plausible as experimental evidence has indicated that dietary restriction may increase mitochondrial turnover [117]. Modelling has also been used to investigate the interaction between caloric restriction and mitochondrial metabolism. For instance Miwa et al. [118] investigated dietary restriction, using computational modelling to support their experimental findings. It was estimated that the liver mitochondria of mice had a significantly reduced median half-life following 3 months dietary restriction, when compared with controls (1.16 days compared with 1.83 days), thus supporting the hypothesis that dietary restriction may promote mitochondrial turnover [118].

Mitochondria have been observed to undertake a complex fusion–fission cycle [119] and computational modelling has also helped improve our understanding of this process. Kowald and Kirkwood [120] used mathematical modelling to argue that fusion is necessary, due to the migration of mitochondrial genes to the nucleus and that mitochondrial fusion is the underlying mechanism regulating the accumulation of mitochondrial mutants with age, while fission may ameliorate this accumulation [120,121]. Mouli et al. [122] determined that during conditions of elevated damage, the selectivity of a fusion event is particularly important as it allows an increase in the frequency of fusion without comprising damaged content removal. The mathematical model developed by Tam et al. [123] demonstrated that low fission–fusion reduced mtDNA mixing resulting in an uneven distribution of mutant mtDNA within mitochondria and increased stochasticity from a mitophagic event. Consequently, clonal expansion of mutant mitochondria became more frequent. The model also predicted that protective retrograde signalling depended on fusion–fission efficiency [123]. The mitochondrial infectious damage adaptation (MIDA) model describes that the decline in the rate of fusion–fission cycling may reflect a systemic adaptation to prolong lifespan by reducing damage spread [124].

### Dysregulation of cellular signalling

#### Target of rapamycin signalling

The target of rapamycin (TOR) protein is a protein kinase that exists in two distinct multi-subunit complexes, mTORC1 and mTORC2 and is capable of sensing nutrient and amino acid availability, growth factor and hormonal signals [125–127]. Depending upon the state of these inputs, the TOR signalling pathway regulates cell growth, autophagy, protein production as well as energy stores around the body. With age, numerous proteins within the mTOR network can become dysregulated [128,129]. Numerous groups have used computational models to attempt to map the kinetics of the system and identify intervention strategies to manage the dysregulation of the network in age-related disorders [130,131]. Kholodenko and colleagues have used multiple mathematical models to investigate cancer biology [132]. Kholodenko and colleagues investigated the response of feedback loops within the mTOR network to different inhibitors gaining insight into the dynamics of the system under different perturbations [133,134]. They along with other groups, have demonstrated that dynamic modelling could be used to understand the effect on both mTOR and related network proteins following a single targeted intervention of a protein within the mTOR network. The main focus of these studies has been on AKT and PTEN and these two proteins are regularly found to be dysregulated with age [132,135]. Mathematical modelling has provided a framework to integrate mTOR to various other cellular processes including cellular senescence [30]. Dalle Pezze et al. [30] integrated a dynamic model of the mTOR network model with a dynamic model of cellular senescence. They identified potential new interventions for attenuating cellular senescence. Due to the strong links between mTOR signalling and insulin signalling, many efforts have been made to create mathematical models of the two combined especially in the area of Type 2 diabetes mellitus (T2DM) [136,137]. Cedersund et al. [138] used such a model to identify a feedback loop between mTORC1 and insulin signalling, which is reduced in T2DM.

#### FOXO signalling

The family of transcription factors known as Forkhead box O proteins (FOXO) are conserved throughout species from *Caenorhabditis elegans* to humans and it has long been known that modulation of these proteins can increase or decrease lifespan [139,140 ]. While FOXO proteins play a key role in aging, there are very few mathematical models focusing on their role in this process. Dynamic models that do focus on aging often include FOXO proteins and their interactions with the mTOR network as part of a model [141,142]. In their model of cellular senescence, Dalle Pezze et al. [30] modulated the levels of FOXO3A activity and assessed the reaction of mitochondrial mass following this activation. The authors showed that an increase in FOXO3A activity reduced mitochondrial size and reduced DNA damage indicating that FOXO3A modulation could play a key role in cellular senescence [30]. Work carried out by Smith and Shanley [143] has modelled in depth the post-translational modifications on FOXO proteins. Building on this, they investigated the effect of ROS on FOXO activation and translocation [143,144]. Their mathematical model showed that at low oxidative stress, FOXO up-regulated the antioxidant defence, whereas under chronic oxidative stress, it is down-regulated.

#### Insulin/Insulin-like growth factor signalling

The insulin and insulin-like growth factor (IGF) signalling (IIS) pathway plays an important role in energy metabolism and growth. Reduced IIS, enhanced insulin sensitivity and reduced plasma IGF-1 have been associated with longevity in invertebrate and murine species [145,146]. To examine the metabolic dynamics associated with insulin resistance (IR), Nogiec et al. [147] developed a flux balance model. This model suggested that the metabolic phenotypes associated with IR are likely due to the dysregulation of several key nodes rather than a single gene defect. The model also demonstrated that dual knockdown of pyruvate dehydrogenase and lipid uptake or lipid/amino acid oxidation reduced ATP synthesis, TCA cycle flux and metabolic flexibility [147].

Disruptions to the IGF pathway are also heavily implicated in the maintenance of health span. For instance the IGF pathway is highly activated in ovarian cancer. To investigate the impact of the IGF system on cell proliferation, Tian and Kreeger (2014) created a kinetic model, which suggested the binding of IGF-binding proteins (IGFPBs) to IGF-1 significantly reduced IGF-1-mediated proliferation and that treatment to block IGF1–IGF1R binding would be more effective at inhibiting cell proliferation, than neutralizing IGF-1 [148]. Additionally, the insulin–TOR–MAPK network model developed by Nijout and Callier [141], which correctly demonstrated that MAPK, active PI3K and GLUT4 responded in a dose-dependent manner to insulin, demonstrated that at lower insulin levels, PTEN knockout increased protein synthesis, and increased insulin sensitivity by GLUT4 activation, consistent with PTENs role as a tumour suppressor.

#### TGF-β signalling

Knowledge of TGF signalling has benefited from expanding on already developed models. Vilar et al. [149] originally developed a concise model to represent the pathway. This model showed receptors as not only transducers of signal but key modulators of a downstream TGF-β response. Shortly after, Schmierer et al. [150] created two models with altered SMAD phosphorylation and nucleocytoplasmic dynamics. Attempting to match both of these models to their experimental data highlighted the importance of correct SMAD dynamics, as only one of the models could accurately fit the data. Zi et al. [151] realized the importance of including the dynamics of both receptors and SMADs, and so created a more comprehensive model that took elements from both Schmierer et al. [150] and an earlier model developed by Zi and Klipp [152], as well as including TGF-β depletion and ligand dynamics. Wegner et al. [153] expanded on elements of all these previous models to include more detailed negative- and positive-feedback mechanisms, allowing them to replicate oscillations seen in experimental data.

TGF-β is known to signal through different type 1 receptors resulting in downstream phosphorylation of either SMAD2/3 or SMAD1/5/8, depending on the particular SMAD-phosphorylated TGF can mediate completely different gene expression signatures. The previous models only examined SMAD2 dynamics, however a previous model also incorporated SMAD1/5/8 and showed that the SMAD7-mediated cross-talk between the two SMAD pathways is important for determining cellular responses [154]. How SMAD signalling changes as we age may be important in the development of a range of diseases. A model detailing the changes in TGF-β receptors over time and the consequential changes in gene expression showed that this contributes to osteoarthritis development during aging [155]. Understanding the receptor dynamics as well as how they change with age is of great importance for many diseases and computational modelling could be of paramount importance to understand this pathway.

#### NF-κB signalling

The nuclear factor-κB (NF-κB) signalling pathway mediates the expression of genes that influence a range of biological processes including immunity, inflammation, cell differentiation and apoptosis, which are activated by a range of stimuli, including infection, ROS and DNA damage [156,157]. Elevated NF-κB has been associated with the onset of several age-related diseases, whereas inhibition of NF-κB has been linked with the delayed onset of age-related diseases in murine models [158]. There are a large number of models representing different aspects of NF-κB signalling e.g. [159,160]. Pogson et al. [161] used agent-based modelling to predict an important role for IκBα–actin interactions, which may be important for NF-κB–IκB complex formation and negative feedback [161,162]. Gong et al. [163] modelled the HMGB1–p53–NF-κB–Ras–Rb network and demonstrated that knockout of A20 destroyed the IκB/NF-κB negative-feedback loop and liberated NF-κB. Elevated NF-κB increased the concentration of cyclin E, which has been associated with cancer proliferation [163]. By using fuzzy logic modelling, Kriete et al. [164] describe that a reduction in NF-κB also improves mitochondrial and biosynthesis functions rapidly. It is important to note that none of these models have explicitly modelled aging. Chronic, low-grade inflammation is an important contributor to human aging and has been termed as inflammaging [165]. Thus, in the future it would be worthwhile adapting or modifying these models to explore this phenonemon.

#### Cytokine signalling

Cytokines are critical in the regulation of inflammatory responses. Their interactions are complex and how they change with age is important in a number of diseases. One of the major cytokines is interleukin-1 (IL-1) and its importance is reflected in the amount of work that has been done on modelling its interactions and changes in IL-1 signalling with age. Proctor et al. [166] developed a model that detailed the interactions between IL-1 and oncostatin M (OSM) and explored how their synergy leads to excessive cartilage destruction. The group also incorporated IL-1 into a model of cartilage aging with multiple other cytokines, to show how changes with age can lead to the development of conditions, in this case cartilage breakdown resulting in [155].

Circadian rhythms can be important in the inflammatory response, changing the level and effect of cytokines, as modelled by [167]. They showed how the levels and effects of IL-1 among others can change over a 24-h light/dark cycle. Incorporating how these rhythms change in an aging model could help explain the role of cytokines in aging and age-related diseases. Cytokines, of course, encompass much more than just IL-1, which is why Baker et al. [168] created a model attempting to replicate an overview of all cytokine interactions in rheumatoid arthritis. Rather than having all cytokines as a separate species in the model, they created a two-variable model encompassing all cytokines into pro- or anti-inflammatory stimulus. Using only these two species they showed a range of possible behaviours that demonstrated how disease states can develop over time.

#### Parathyroid hormone signalling

Bone remodelling is vital for maintenance of healthy bone as it allows removal of old bone, as well as repair from micro-fractures making sure it remains strong and healthy throughout life. This process was modelled extensively in Lemaire et al. [169], showing a vital role for parathyroid hormone (PTH). Matching simulations to experimental data they showed how aging effects (such as oestrogen deficiency) can alter bone remodelling, even in conjunction with currently used or potential drug interventions.

Administration of PTH is used to combat bone loss but it must be applied intermittently as constant exposure can cause bone loss. A computation model was developed to examine the mechanisms of the opposing effects of PTH [170]. The model demonstrated how PTH could cause both bone growth and loss, leading to a novel hypothesis that it was mainly due to the effect of PTH on osteoclast (cells that remove bone) activation. Expanding on this, another computational study looked at the effect of PTH and loading on aging-related bone loss [171]. The importance of precise PTH cycles in addition to regular loading is demonstrated in this model. In addition, the model was used to examine the effect of PTH treatments with age, their results agreeing with the conclusion in [170].

Geometric regulation has not been incorporated into these models. However, Pivonka et al. [172] demonstrated its importance in bone remodelling and early development of osteoporosis, suggesting any future models of age-related bone loss should be aware of its effect on PTH and loading.

#### DNA methylation dynamics and computational modelling

There is growing evidence that DNA methylation status and intrinsic aging are inexorably correlated. This view is supported by a wealth of experimental evidence. Most strikingly, the computational work of Horvath, S. [173] who used methylation data sets to pinpoint an ‘epigenetic clock’ whose time is governed by methylation changes within several hundred CpGs, a CpG being a dinucleotide consisting of a deoxycytidine followed by a deoxyguanidine, with the ‘p’ indicating the phosphate group between these nucleotides. More recently, others such as Curtius et al. [174] used methylation data and Bayesian modelling to estimate patient-specific disease onset times in what they suggest is a “molecular clock which can infer specific tissue age in patients with Barrett’s Oesophagus”. These findings are intriguing as epigenetic mechanisms such as DNA methylation are modifiable and offer the possibility that aging may be reversible or at very least malleable. However, the biochemical and molecular processes involved in the regulation of DNA methylation events are multifaceted and exceptionally complex. Gaining a deeper understanding of these processes is challenging. For instance the enzyme-mediated events that are responsible for the addition and removal of methyl groups to CpG dinucleotide intersect with folate one carbon metabolism (FOCM) [175]. Moreover, it has been shown that both FOCM and DNA methylation are affected by other factors associated with aging. For instance both FOCM and DNA methylation are affected by oxidative stress [176–178]. In addition, the activity of DNA methyltransferase 1, the key enzyme responsible post-replication for transferring methyl groups to the DNA molecule has been shown to be influenced by sirtuin-1 (Sirt1) [179]. In recent years, a number of models have been used to mechanistically represent DNA methylation, and these could be adapted to focus on cross-talk between DNA methylation and other elements of cellular aging. For example a recent model that was constructed using partial differential equations was able to fully represent the full suite of DNA methylation/demethylation reactions and was used as a tool for predicting haematological malignancies [180]. More specific to intrinsic aging, Przybilla et al. [181] used a stochastic model to explore age-related changes in DNA methylation within stem cells and simulations suggesting homing at stem-cell niches retarded epigenetic aging.

#### miRNAs and modelling

miRNAs are evolutionarily conserved post-transcriptional non-coding gene regulators, which operate by inducing mRNA degradation or translational repression in a site sequence specific manner [182]. Mathematical modelling has played a unique role in helping to unravel the dynamics, which underpins their behaviour. Specifically modelling has helped to identify feedback and feedforward loops in miRNA-mediated networks and has revealed interactions among miRNAs during the regulation of genes (reviewed in [183]). Although models have mainly focused on cancer, many of the biological processes that have been modelled have also been implicated with intrinsic aging. For instance Lai et al. [184] used a computational model based on the p53/Sirt1 signalling pathway to explore the regulatory effect of *miR-34a* on p53 through its impact on Sirt1. This model demonstrated inhibition of p53 activity due to Sirt1 up-regulation could be mitigated by up-regulating *miR-34a* expression. Given the proposed role of Sirt1 in aging, it would be worthwhile adapting a model such as this to explore this relationship further to assess the implications for aging. Models have also explored other aspects of cellular aging. For example the model by Xue et al. [185] integrated *miR-21* and *miR-146* expression into a signalling pathway to create a mode of the inflammatory response. Output from the model showed that negative feedback provided by *miR-21* modulated the oscillatory behaviour of NF-κB and IL-6 activity. In addition, the model demonstrated negative feedback by *miR-146* dampens the oscillations of NF-κB and IL-6, indicating these are mediators of this process.

#### Tissue regeneration

A decline in tissue regeneration is another important factor in cellular aging and is considered to be mainly due to a depletion or loss of function of stem cells and altered intercellular communication [186,187]. There are multiple molecular mechanisms contributing to this decline including an accumulation of DNA damage, telomere shortening, epigenetic changes and loss of protein homoeostasis. These mechanisms cause cellular damage and lead to cellular responses, which initially reduce the damage but if activated chronically can produce deleterious effects themselves [186]. Finally, the chronic activation of cellular responses to damage leads to the decline in tissue function due to stem cell exhaustion and disruption of intercellular signalling [186]. For example muscle regeneration after injury requires differentiation of satellite cells but with age there is a gradual decline in the response to damage signals. As a multitude of overlapping processes is involved in tissue regeneration, computational modelling is an ideal tool for investigating their interactions and it offers the potential of helping to isolate those mechanisms which are fundamental to this phenomenon. To date, a number of worthwhile models have been developed that touch on various aspects of this biological system. For instance stem cell dynamics have been modelled ubiquitously, however to our knowledge only two models of this nature have specifically centred on aging. These are the models by Przybilla et al. [181] that examined the role of age-related DNA methylation changes (as discussed previously), and Duscher et al. [188] that modelled the effect of aging on MSC population dynamics and showed that an age-related depletion in a subpopulation of progenitor cells impaired the formation of new blood vessels. If these models and elements of the other models we have discussed could be integrated together, with processes such as disrupted intracellular communication and the concomitant increase in the inflammatory response due to the secretion of pro-inflammatory cytokines by senescent cells, together with the activation of NF-κB, this would result in a comprehensive model. This model would be more than capable of representing and exploring the dynamics of tissue regeneration in greater depth. In the next section, we will discuss the technical challenges associated with combining models and how this can be overcome in the future.

### Opportunities and challenges for the future

#### Integration of mechanisms

Despite the insights that computational modelling efforts have contributed to understanding of aging, *in silico* representations of biological systems are constrained to specific experimental models, specific canonical pathways or a specific stimulus. However, recent efforts have taken on the challenge to expand the models to include cross-talk and multiple input stimuli in an attempt to more faithfully recreate the complex nature of the underlying signalling networks [30,166,189–191].

The practice of model integration is not uncommon. For example Markevich et al. [192] expanded a previously developed model of the electron transport chain, and Gauthier et al. [114] combined a model of mitochondrial metabolism with an antioxidant model to produce a comprehensive virtual mitochondrion. However, it has only been relatively recently that the tools have become available [193,194] to standardize and automate the model integration process through the identification of shared model variables via semantic analysis of SBML [195]. It is worth noting that most of such automated methods of model integration operate for networks of the same scale (e.g. subcellular pathways) and of the same mathematical framework (e.g. ODE models). Integrating models beyond scales and computational frameworks is an avenue of current work and challenges [196].

The computational power needed to cost effectively simulate larger integrated models is a major hurdle against the effort to construct more complex networks. An elegant example of how this problem can be addressed can be seen in the abstraction of the mTOR network by Dalle Pezze et al. [30]. The authors used a previously developed model of mTOR and abstracted it to a network motif while retaining the key observables to encode system behaviour and then integrated it with abstracted representations of the DNA damage and stress responses. The increasing number of *in silico* models becoming available will offer a greater opportunity for model integration in response to new findings in the scientific literature.

#### Multi-scale models

From our discussion of aging, it is clearly an exceptionally complex process with a multitude of overlapping mechanisms and networks. Despite this, many of the models we have discussed centre on very specific processes, however, as of yet there is no fully integrated model that encapsulates all the current knowledge about aging. It is imperative that this issue is addressed, as a fully integrated model will lead to a deeper understanding of aging [197,198]. To do this, several computational challenges need to be addressed. Firstly, the factors associated with aging operate over widely different timescales and in many instances it is appropriate to assume spatial homogeneity. For example when modelling a biochemical/physiological network, a deterministic-based ODE model is suitable. However, this assumption is not always valid, especially when considering molecular systems where noise is an issue due to the inherent statistical mechanical fluctuations in the binding and discrete dynamics of the molecules involved in the reactions. Therefore, stochastic models are more suited to this type of system. It is necessary to develop computational approaches capable of integrating these theoretical approaches in a meaningful way. Recently, several worthwhile examples of hybrid computational approaches have been developed to address this problem. In an eloquent hybrid model of cell-cycle regulation, Singhania et al. [199] utilized continuous ODEs and discrete Boolean networks. The model tracked cyclin levels by using piecewise linear differential equations, with cyclin synthesis and degradation modulated by discrete variables. More specific to aging, Kriete et al. [164] developed a rule-based cell systems model. This model of cellular aging incorporated the mTOR pathway coupled with mitochondrial homoeostasis and the NF-κB pathway. In addition to these examples, the computational systems biology community has proposed several solutions to the challenge of multi-scale modelling. Sütterlin et al. [200] developed a novel software workflow (EPISIM) for the semantic integration of SBML-encased models. More recently, Somogyi et al. [201] developed libRoadRunner – a Python-based application which supports large-scale problems for SBML-encoded models, including multi-module modelling. Continued progress of this nature in the field of computational biology will be a significant benefit to those using computational models to investigate the complexities of aging.

## Conclusions

In this article, we have given an overview of the currently available models that are relevant for increasing our understanding of the molecular mechanisms of aging. The majority of models focus on particular mechanisms and many of the earlier models were constructed in such a way as to make it difficult to modify or integrate. With the advent of new systems biology tools and modelling standards such as SBML, computer models have become much more adaptable and there is now a very useful pool of models available. This will hopefully allow for more integrative models to be constructed in the future, as it is now clear that biological aging is not driven by an individual process, but involves a complex interplay of many different mechanisms.

## Abbreviations

AD: Alzheimer’s disease
CMA: chaperone-mediated autophagy
CpG: 5'-C-phosphate-G-3'
DDR: DNA damage response
FOCM: folate one carbon metabolism
FOXO: Forkhead box O proteins
GLUT4: Glucose transporter type 4
HSF-1: heat shock factor-1
Hsp70: heat shock protein 70
Hsp90: heat shock protein 90
IGF: insulin-like growth factor
IIS: insulin/IGF signalling
IL-1: interleukin-1
IR: insulin resistance
mTORC: mammalian target of rapamycin complex
ODE: ordinary differential equation
PCNA: proliferating cell nuclear antigen
PD: Parkinson’s disease
PI3K: Phosphatidylinositol-4,5-bisphosphate 3-kinase
PTEN: phosphatase and tensin homolog
PTH: parathyroid hormone
PySB: Pytbon framework for Systems Biology modelling
ROS: reactive oxygen species
SBML: systems biology markup language
Sirt1: sirtuin-1
TCA: tricarboxylic acid
TGF: transforming growth factor
TOR: target of rapamycin
T2DM: Type 2 diabetes mellitus
